# Investigating the Psychometric Properties of the Persian Version of the Revised Illness Perception Questionnaire in a Sample of Patients With Multiple Sclerosis

**DOI:** 10.1002/brb3.70821

**Published:** 2025-09-02

**Authors:** Ali Bagheri, Maryam Amini‐Fasakhoudi, Mohammad Ali Sahraian, Abbas Masjedi‐Arani, Mohadeseh Hashemi, Fatemeh Serjouie, Amir Sam Kianimoghadam

**Affiliations:** ^1^ Department of Clinical Psychology, School of Medicine Shahid Beheshti University of Medical Sciences Tehran Iran; ^2^ Multiple Sclerosis Research Center, Neuroscience Institute Tehran University of Medical Sciences Tehran Iran; ^3^ Department of Psychology University of Social Welfare and Rehabilitation Sciences Tehran Iran

**Keywords:** Illness Perception Questionnaire, multiple sclerosis, psychometric properties

## Abstract

**Introduction:**

Illness perception significantly influences treatment adherence and quality of life among patients with multiple sclerosis (MS). Despite the widespread use of the Revised Illness Perception Questionnaire (IPQ‐R) in different languages, a validated Persian version tailored for MS patients was lacking.

**Methods:**

A cross‐sectional study was conducted with 400 Persian‐speaking MS patients recruited from the MS Society of Iran between December 2023 and April 2024. The IPQ‐R was translated and culturally adapted through standard forward–backward translation procedures. Validity was assessed through face, content, and construct validity (using both confirmatory factor analysis [CFA] and exploratory factor analysis [EFA]). Reliability was evaluated using Cronbach's alpha and composite reliability (CR).

**Results:**

The Persian IPQ‐R showed strong face and content validity, with all items achieving acceptable content validity ratio (CVR) and content validity index (CVI) scores. CFA supported the original seven‐factor model (*χ* ^2^/df = 1.048, CFI = 0.998, RMSEA = 0.011). EFA of the cause subscale revealed four interpretable dimensions explaining 75.6% of variance. Most subscales demonstrated excellent internal consistency (*α* = 0.839–0.962), whereas the identity subscale showed lower reliability (*α* = 0.590). Convergent and discriminant validity were confirmed through average variance extracted (AVE) and Heterotrait–Monotrait (HTMT) indices.

**Conclusion:**

This study revealed that the Persian version of the IPQ‐R is a reliable and valid instrument for assessing illness perceptions in Iranian MS patients. It may serve as a valuable tool in clinical and research settings to evaluate patient beliefs and guide personalized care strategies.

## Graphical abstract

The Persian version of the IPQ‐R demonstrated excellent validity and reliability among 400 Iranian MS patients. Confirmatory and exploratory analyses supported the original seven‐factor model and revealed four meaningful cause subscales. This validated tool enables tailored assessment of illness beliefs in Persian‐speaking MS populations.

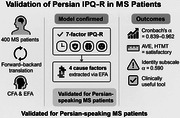

## 1 Introduction

Multiple sclerosis (MS) is an immune‐mediated chronic disorder of the central nervous system (CNS) characterized by myelin damage and axonal degeneration. MS represents a leading cause of neurological disability in young adults globally, predominantly affecting individuals between 20 and 40 years of age. A notable sex disparity exists, with female‐to‐male prevalence ratios ranging from approximately 2:1 to 3:1 depending on geographic region, indicating a higher incidence among women. Approximately one person is diagnosed with MS every 5 min worldwide, with a mean age of onset around 32 years. This contributes to the estimated 2.8 million individuals currently living with the disease (Khutsishvili et al. 2024). Despite its location within a region historically considered to have a low risk of MS (geographical coordinates: 32° N, 53° E), Iran is currently recognized as a country with high MS prevalence. In 2020, the pooled prevalence of MS in Iran was reported to be 0.001 (100 per 100,000), a threefold increase compared to estimates by Azami et al. in 2019, indicating a substantial rise in MS incidence within the country (Mirmosayyeb et al. 2022). MS symptoms result from myelin loss and, depending on the location of plaques, may include weakness, dizziness, muscle spasms, numbness, gait disturbances, emotional changes, visual impairments, fatigue, bladder dysfunction, paralysis, ataxia, and progressive cognitive decline. Additionally, these symptoms are often accompanied by various psychiatric manifestations, such as depression and anxiety (Ziaie et al. 2024).

Although MS is not curable, disease‐modifying therapies (DMTs) are available and effective in managing the disease by reducing relapses and slowing progression. However, a significant challenge remains in patient adherence to these treatments, with studies showing that a substantial proportion of MS patients discontinue DMTs prematurely. This poor adherence can be attributed to several factors, including the uncontrollable nature of MS symptoms, prolonged remission periods, side effects of treatment, and lack of visible improvement (Wilski et al. 2019). Addressing these factors by modifying patients’ illness perceptions and treatment beliefs is crucial for improving treatment adherence and, consequently, their quality of life.

The Common‐Sense Model of Self‐Regulation (CSM) explains how individuals perceive and respond to health threats. According to this model, when faced with illness, people form beliefs about their condition and treatment, which guide their coping strategies and help restore health. The CSM proposes that illness‐related stimuli trigger both cognitive (e.g., symptoms, causes, and consequences) and emotional (e.g., worry and distress) responses. These responses unfold in three stages: (1) constructing illness representations (cognitive and emotional); (2) implementing coping strategies based on these representations; and (3) evaluating the effectiveness of these strategies, which then feed back to modify or reinforce the initial representations (Benyamini and Karademas 2019; Hagger and Orbell 2022).

The Illness Perception Questionnaire (IPQ) was developed to assess the constructs of the CSM. Initially, it included five dimensions: identity, cause, timeline, consequences, and control/treatment (Weinman et al. 1996). Later, the revised IPQ (IPQ‐R) was created to improve its psychometric properties by adding emotional/affective aspects of the illness and measuring illness coherence (understanding of the diagnosis). The IPQ‐R now contains nine dimensions: cause, identity, acute/chronic timeline, cyclical timeline, consequences, personal control, treatment control, illness coherence, and emotional representation (Moss‐Morris et al. 2002).

Although there are other tools available for assessing illness perceptions in various populations (e.g., the Brief IPQ) (Broadbent et al. 2006), these instruments have not been adequately adapted or validated for Persian‐speaking MS patients. Other tools such as the Multidimensional Health Locus of Control (MHLC) (Wallston and Wallston 1981) and the Self‐Regulation Questionnaire (SRQ) (Schwarzer and Schmitz 2005) are also commonly used to measure beliefs and perceptions about illness. However, these tools do not capture the full range of illness representations as outlined by Leventhal's CSM, particularly missing the emotional aspects of illness and the coherence of the illness experience. Additionally, they are not specific to MS patients, limiting their application in this context.

Although the original IPQ‐R is available in English and has been validated with MS patients in English‐speaking populations, Persian is the official language of Iran. Therefore, to ensure that Persian‐speaking MS patients can understand the items in the original IPQ‐R, it is necessary to translate the questionnaire into Persian and assess its validity and reliability within this population. To the best of the authors’ knowledge, no validated and reliable Persian‐language IPQ currently exists for assessing illness understanding among MS patients in Iran. Thus, the aim of this study is to evaluate the validity and reliability of the Persian version of the IPQ‐R in a sample of MS patients, with the objective of better characterizing their illness perceptions.

## 2 Methods

### 2.1 Participants and Procedures

This cross‐sectional study was conducted from December 2023 to April 2024. A convenience sample of 400 individuals with MS was recruited from those attending the MS Society of Iran, providing a sample size sufficient for factor analysis. Adhering to Kline's recommendations, maintaining a participant‐to‐item ratio of 5:1–10:1 is crucial in factor analysis. Furthermore, Kline (2013) suggests a minimum sample size of 200 participants for confirmatory factor analysis (CFA). The modified IPQ‐R comprised 74 items. Consequently, the minimum required sample size for this study was determined to be 370 patients with MS. Therefore, the present study, with a sample size of 400 MS patients, exceeded the suggested minimum requirement.

Inclusion criteria included age between 18 and 55 years, a diagnosis of MS by a neurologist for at least 6 months, the ability to read and write, the capacity to independently complete the questionnaire without cognitive or physical impairments, and no severe physical disability (EDSS score ≤ 6). Exclusion criteria included the inability of cognitively impaired individuals to complete the questionnaire (e.g., due to cognitive issues or comprehension failure), severe anxiety or distress while completing the questionnaire that prevents appropriate responses, and reluctance to participate or withdrawal at any stage of the study. These criteria ensured the validity and reliability of the data collected, which was essential for assessing the psychometric properties of the Persian version of the IPQ‐R.

Following approval of the study protocol by the Research Ethics Committee of the School of Medicine, Shahid Beheshti University of Medical Sciences (Ethics Code: IR.SBMU.MSP.REC.1403.152), trained research assistants identified eligible patients among those awaiting their appointments and approached them while distributing study materials in the outpatient clinic. The researchers briefly introduced themselves as researchers and provided patients with details about the study (including its purpose and main content), asking if they were willing to participate in the survey. Participants were assured that their refusal would not affect their medical care at the hospital, and that they could withdraw at any time during the survey without being questioned. Participants were also assured of the confidentiality of the information they provided to the researchers and their anonymity. If a patient agreed to participate, they signed an article consent form before completing the survey. A total of 470 patients were approached, of whom 70 declined to participate, resulting in 400 completed surveys (response rate: 85%). To mitigate biases arising from reduced attention or motivation during the assessment, the order of questionnaire presentation was randomly varied. On average, participants spent 30 min completing the questionnaires.

### 2.2 Translation and Cross‐Cultural Adaptation Processes

After obtaining consent from the original author, the translation process commenced, structured into five distinct phases. In the first phase, the English version of the IPQ‐R was selected as the source for translation. The second phase involved the recruitment of two translators, both fluent in English and native Persian speakers, to translate the questionnaire from English to Persian. One translator was a health psychologist, whereas the other specialized in language with no prior exposure to concepts related to illness perception. Both translators worked autonomously, with instructions to remain as faithful as possible to the original English text. By the end of this phase, two separate Persian versions of the questionnaire were produced, one by each translator.

In the third phase, a review team consisting of a neurologist and two clinical psychologists evaluated the two Persian versions generated in the second phase and integrated them into a singular cohesive version. This integration process utilized terminology that accurately captured the essence of the original English text. Moving to the fourth phase, two English‐speaking translators, who were unaware of the original questionnaire content, were engaged to back‐translate the consolidated Persian version into English. These translators, with no familiarity with the measurement constructs of the questionnaire, also worked independently, resulting in two English back‐translations of the questionnaire.

Finally, in the fifth phase, the translation team evaluated the two English back‐translations produced in the fourth phase and synthesized them into a single finalized version, culminating in the completion of the translated questionnaire.

### 2.3 Instruments

#### 2.3.1 Sociodemographic Questionnaire

A researcher‐designed demographic questionnaire was employed to gather information on the participants’ demographic characteristics, including age, gender, educational attainment, and marital status.

#### 2.3.2 Revised Illness Perception Questionnaire

The IPQ‐R is a widely used tool for assessing individuals’ perceptions of their illness. It consists of nine subscales, each addressing different aspects of illness perception. The subscales included in the Persian version of the IPQ‐R are as follows:

1. Identity: This subscale includes 18 symptoms (such as pain, nausea, fatigue, and others). Participants are first asked whether they have ever experienced each symptom (with responses coded as 1 for “yes” and 0 for “no”). Subsequently, they are asked whether they consider these symptoms as part of their illness. If the participant indicated “yes” for a symptom in the first set, they can then indicate “yes” or “no” in the second set, indicating whether they associate the symptom with their illness. If the symptom was not experienced initially, the second response is automatically “no.” The relationship between the two sets of questions is as follows:

Questions 1–18 (symptoms experienced) correlate with Questions 19–36 (symptoms attributed to illness).

1. Timeline acute/chronic: This subscale consists of Questions 37–41 and 54. It assesses individuals’ perceptions of their illness as either acute or chronic. Responses are recorded using a 5‐point Likert scale: 1 (Strongly Disagree), 2 (Disagree), 3 (Neutral), 4 (Agree), and 5 (Strongly Agree).
2. Timeline cyclical: Comprising Questions 65–68, this subscale measures the extent to which individuals perceive their illness as cyclical or episodic. Like the timeline acute/chronic subscale, responses are recorded on a 5‐point Likert scale.
3. Consequences: This subscale (Questions 42–47) evaluates the perceived consequences of the illness. It uses a 5‐point Likert scale, where responses range from 1 (Strongly Disagree) to 5 (Strongly Agree).
4. Personal control: Comprising Questions 48–53, this subscale assesses the perceived personal control over the illness. Responses are provided on a 5‐point Likert scale, ranging from 1 (Strongly Disagree) to 5 (Strongly Agree).
5. Treatment control: This subscale includes Questions 55–59 and assesses participants’ perceptions of the control they have over their treatment. It uses the same 5‐point Likert scale.
6. Illness coherence: The subscale measuring the understanding of the illness consists of Questions 60–64. It evaluates the clarity with which individuals understand the nature of their illness. Responses are again given using a 5‐point Likert scale.
7. Emotional representation: This subscale, including Questions 69–74, assesses emotional responses to illness. Participants respond using a 5‐point Likert scale ranging from 1 (Strongly Disagree) to 5 (Strongly Agree).
8. Cause: The final subscale, which includes Questions 75–92, assesses the perceived causes of illness. Like other subscales, responses are given on a 5‐point Likert scale.

The IPQ‐R has been widely used and demonstrates robust psychometric properties, including high internal consistency and test–retest reliability. Construct validity has been confirmed through CFA, with adequate fit indices supporting the seven‐factor structure initially proposed by Moss‐Morris et al. (2002). The subscales are designed to assess a wide range of illness perceptions, and although the tool has shown strong reliability and validity in various cultural contexts (Hagger et al. 2017), the Persian version of the IPQ‐R has not yet undergone full normative validation. Therefore, the present study seeks to establish its validity and reliability in the Persian‐speaking population.

### 2.4 Validity

#### 2.4.1 Face Validity

To assess the face validity of the IPQ‐R, both quantitative and qualitative evaluation methods were employed. For the quantitative assessment of face validity, the items of the developed instrument were rated using a 5‐point Likert scale ranging from very important (Hagger and Orbell 2022) to not important (Khutsishvili et al. 2024; Polit and Beck 2020). The proposed questionnaire was then administered to a panel of seven experts, consisting of two PhDs in nursing, two neurologists, and three health psychologists, none of whom had previously participated in the specialist panel. The frequency with which experts assigned scores of 4 and 5 to each item was analyzed, along with the total score and average score for each item (reflecting importance). Following this, the impact score for each item was computed using the formula (impact = frequency × importance) (Polit and Beck 2020). An impact score of 1.5 or higher was deemed acceptable for each item (Polit and Beck 2020).

Furthermore, for the qualitative assessment of face validity, a convenience sample consisting of 60 individuals from the target population was utilized. These participants were invited to provide feedback regarding the difficulty of the items, the clarity of the terminology and phrases, and the relevance and suitability of the content, as well as any ambiguities or possible misinterpretations associated with the wording (Polit and Beck 2020).

#### 2.4.2 Content Validity

The assessment of content validity utilized both quantitative and qualitative approaches. On the quantitative side, content validity ratio (CVR) and content validity index (CVI) were employed, as recommended by Polit and Beck. A translated version of the questionnaire was distributed to seven experts, including two PhDs in nursing, two neurologists, and three health psychologists, who evaluated it according to three criteria: “relevance,” “clarity,” and “simplicity.” The relevance was rated on a 4‐point scale from 1 (“not relevant”) to 4 (“completely relevant”). The simplicity of the items was similarly assessed on a 4‐point scale, ranging from 1 (“not simple”) to 4 (“completely simple”), whereas clarity was judged on the same 4‐point scale from 1 (“not clear”) to 4 (“completely clear”). The CVI was determined by dividing the number of experts who rated an item as either a 3 or a 4 by the total number of experts, yielding individual scores for each item (Leung et al. 2018; Woo 2017).

To compute the CVR, feedback was sought from the same group of seven specialists regarding the relevance of the content. After outlining the objectives of the questionnaire and providing operational definitions pertinent to the items, the experts were prompted to categorize each item using a 3‐point Likert scale: “the item is useful,” “the item is useful but not essential,” and “the item is not useful.” The results for all items in the IPQ‐R were then compiled and analyzed following the Lavish methodology (Binti Hassan 2018; Polit and Beck 2020).

Qualitative content validity was assessed by a panel of seven specialists (two nurses, two neurologists, and three health psychologists), who offered their perspectives on the suitability of the content organization, appropriateness of language use, adherence to grammatical conventions, and the appropriateness of the scales and their accompanying instructions (Polit and Beck 2020).

#### 2.4.3 Construct Validity

Construct validity of the Persian version of the IPQ‐R was evaluated through a multi‐method approach. In the first step, given that the subscales of timeline acute/chronic, timeline cyclical, consequences, personal control, treatment control, illness coherence, and emotional representation are based on a well‐established seven‐factor theoretical framework derived from the CSM, CFA was employed to examine the extent to which empirical data aligned with this theoretical structure (Moss‐Morris et al. 2002). Each item was restricted to load on its corresponding latent factor, and all latent constructs were allowed to correlate (Moss‐Morris et al. 2002). The CFA was conducted using the maximum likelihood estimation method, which is appropriate under the assumption of approximately normal distribution and provides efficient and unbiased estimates (Kline 2023). Univariate normality was evaluated through skewness and kurtosis, with values between −2 and +2 for skewness and −10 to +10 for kurtosis considered acceptable (Kline 2023). Multivariate normality was assessed using Mahalanobis distance, where values exceeding the chi‐square critical threshold indicated potential outliers (Tabachnick and Fidell 2013). Model fit was assessed using chi‐square (*χ* ^2^), CFI, TLI, RMSEA (with 90% CI), and SRMR. Acceptable fit criteria were *χ* ^2^/df ≤ 5 (excellent if ≤3), CFI and TLI ≥ 0.90 (excellent if ≥0.95), RMSEA ≤ 0.08 (excellent if ≤0.06), and SRMR ≤ 0.08 (Hu and Bentler 1999; Hair et al. 2021). All analyses were conducted using SPSS 26 and AMOS 24.

The identity subscale of the IPQ‐R is conceptually and methodologically distinct from the other subscales. It consists of a checklist of symptoms with dichotomous (yes/no) responses, indicating whether respondents associate each symptom with their illness. Unlike the other subscales, which are based on Likert‐scale items designed to measure latent constructs, the identity subscale does not assume a unidimensional latent factor structure. Therefore, it is not appropriate for analysis using CFA or exploratory factor analysis (EFA). Furthermore, the pattern of symptom attribution captured by the identity subscale may vary considerably depending on the nature of the illness, which further limits the applicability of a fixed factor structure. In line with the original standardization study of the IPQ‐R and established psychometric conventions, alternative methods have been employed to assess the structural validity of this subscale (Moss‐Morris et al. 2002). First, a paired sample *t*‐test was conducted to compare the number of symptoms participants reported experiencing in general with the number attributed specifically to MS. A significant difference between these two sets supports the conceptual distinction and reinforces the unique nature of the illness identity construct (Broadbent et al. 2006). Second, a frequency analysis of symptom endorsement was performed to examine internal response patterns. High frequencies of endorsement for specific symptoms provided additional evidence for the structural coherence of the subscale (Lau and Hartman 2017).

Following recommendations by the original IPQ‐R developers, the factor structure of the cause subscale was empirically evaluated using EFA, as the structure may differ depending on illness context (Moss‐Morris et al. 2002). Maximum likelihood extraction with Varimax rotation was employed to identify the underlying latent constructs, assuming orthogonal (uncorrelated) factors. Prior to analysis, the assumption of approximate univariate normality was assessed through the examination of skewness and kurtosis values. Skewness values between −2 and +2 and kurtosis values between −10 and +10 were considered acceptable indicators of normality (Kline 2023). Multivariate normality was evaluated using Mahalanobis distance, with values exceeding the critical chi‐square threshold (based on the number of observed variables as degrees of freedom) considered potential multivariate outliers (Tabachnick and Fidell 2013). Suitability for EFA was confirmed using the Kaiser–Meyer–Olkin (KMO) measure and Bartlett's test of sphericity (Field 2018). Factors were extracted on the basis of eigenvalues >1.0 and scree plot inspection. Items with loadings ≥0.40 were retained and assigned to factors. In the case of cross‐loading, the item was assigned to the factor where the squared loading was at least 50% greater than other squared loadings (Tabachnick and Fidell 2013). Communalities were examined to assess the proportion of each item's variance explained by the extracted factors. Items with very low communalities were considered for exclusion, whereas estimates exceeding 1.0 were interpreted cautiously due to potential artifacts in the estimation process.

Convergent validity was assessed through inter‐correlations among IPQ‐R subscales. On the basis of Cohen's classification, correlations of *r* = 0.10–0.29 were considered weak, *r* = 0.30–0.49 moderate, and *r* ≥ 0.50 strong (Cohen 1988). Moderate‐to‐strong correlations among conceptually related subscales provided evidence of convergent validity (Zhang et al. 2023). To further assess convergent validity at the latent construct level, the average variance extracted (AVE) was calculated for each IPQ‐R subscale. An AVE ≥ 0.50 was considered adequate, indicating that a construct explains at least 50% of the variance of its indicators (Hair et al. 2021; Fornell and Larcker 1981).

Discriminant validity was evaluated to determine whether the IPQ‐R subscales represent distinct theoretical constructs (Moss‐Morris et al. 2002). Pearson correlation coefficients were calculated among the subscales of the IPQ‐R to assess whether the correlations between theoretically unrelated constructs are lower than those between conceptually related subscales. This approach helps to ensure that the subscales are measuring distinct constructs and supports discriminant validity (Fornell and Larcker 1981).

Additionally, the Heterotrait–Monotrait (HTMT) ratio was calculated to further assess discriminant validity. HTMT estimates the ratio of between‐construct correlations (heterotrait–heteromethod) to within‐construct correlations (monotrait–heteromethod) and is considered a superior alternative to classic criteria such as the Fornell–Larcker criterion. An HTMT value below the conservative threshold of 0.85 indicates that the constructs are empirically distinct and not excessively correlated. This method accounts for measurement error and is especially useful when evaluating structurally similar constructs (Henseler et al. 2015).

### 2.5 Reliability

The reliability of the Persian version of the IPQ‐R was assessed using two complementary approaches: internal consistency and composite reliability (CR). These analyses were conducted separately for different subscales based on the type of psychometric model applied (CFA vs. EFA). Internal consistency was evaluated using Cronbach's alpha coefficient for each IPQ‐R subscale. Alpha values greater than 0.70 were interpreted as acceptable, whereas values of 0.80 and above were considered good to excellent (Cho and Kim 2015; Tavakol and Dennick 2011). Internal consistency was assessed for all subscales, including those examined using EFA (e.g., identity and cause).

CR was computed only for the subscales evaluated with CFA (i.e., timeline acute/chronic, timeline cyclical, consequences, personal control, treatment control, illness coherence, and emotional representation), because CR is a model‐dependent index that relies on the factor loadings and error variances estimated within a specified measurement model. For subscales assessed via EFA, CR was not reported, as the absence of a predefined structural model and measurement error estimation makes the use of CR less appropriate in this context (Hair et al. 2021; Kline 2023). CR values ≥0.70 were considered acceptable, indicating sufficient internal consistency among the observed indicators (Hair et al. 2022).

## 3 Results

### 3.1 Characteristics of the Participants

The majority of the 400 participants were middle‐aged (mean = 39, SD = 10.2), women, single, and held a bachelor's degree or lower. Detailed sociodemographic characteristics are presented in Table 1.

**TABLE 1:** Sociodemographic characteristics of the participants (*N* = 400).

Quantitative variable	Mean (SD)
Age	39 (10.2)
Qualitative variables	*N* (%)
Education	
High school diploma or lower	87 (21.8)
Associate's degree	78 (19.5)
Bachelor's degree	86 (21.5)
Master's degree	71 (17.8)
Doctoral degree	78 (19.5)
Gender	
Female	217 (54.3)
Male	183 (45.8)
Marital status	
Marital	186 (46.5)
Single	214 (53.5)

### 3.2 Validity

#### 3.2.1 Face Validity

All 74 items were approved by both the expert panel and participants. Quantitative face validity, presented in Table 2, revealed that all items had impact scores greater than 1.5. In addition, qualitative feedback confirmed that the items were clear, relevant, and comprehensible, with no reported issues regarding ambiguity or appropriateness. Therefore, all items were retained for subsequent validation phases.

**TABLE 2:** Content validity ratios (CVR), content validity indices (CVI), and impact scores for each item of the Revised Illness Perception Questionnaire (IPQ‐R).

Item	CVR (necessity)	CVI (relevance)	CVI (clarity)	CVI (simplicity)	CVI (total)	Impact score
**1. Pain**	0.87	0.84	0.88	0.89	0.87	3.0
**2. Sore throat**	0.84	0.83	0.92	0.93	0.89	2.9
**3. Nausea**	0.84	0.83	0.84	0.88	0.85	3.6
**4. Breathlessness**	0.85	0.82	0.9	0.83	0.85	3.2
**5. Weight loss**	0.88	0.85	0.83	0.83	0.84	2.8
**6. Fatigue**	0.94	0.91	0.89	0.91	0.9	2.9
**7. Stiff joints**	0.87	0.84	0.83	0.86	0.84	2.8
**8. Sore eyes**	0.93	0.92	0.83	0.89	0.88	3.9
**9. Wheeziness**	0.83	0.93	0.82	0.93	0.89	3.0
**10. Headaches**	0.9	0.85	0.89	0.92	0.89	3.6
**11. Upset stomach**	0.9	0.93	0.86	0.94	0.91	3.2
**12. Sleep difficulties**	0.87	0.91	0.84	0.87	0.87	3.3
**13. Dizziness**	0.88	0.9	0.87	0.85	0.87	3.6
**14. Loss of strength**	0.95	0.91	0.9	0.86	0.89	3.6
**15. Speech distortion**	0.95	0.86	0.82	0.83	0.84	3.8
**16. Bladder problems**	0.97	0.9	0.92	0.91	0.91	3.1
**17. Clumsiness**	0.93	0.84	0.88	0.85	0.86	3.2
**18. Numbness**	0.88	0.94	0.82	0.9	0.89	3.2
**19. My illness will last a short time**	0.86	0.89	0.93	0.84	0.89	3.6
**20. My illness is likely to be permanent rather than temporary**	0.91	0.83	0.92	0.87	0.87	3.7
**21. My illness will last for a long time**	0.86	0.85	0.92	0.89	0.89	3.8
**22. This illness will pass quickly**	0.84	0.95	0.87	0.88	0.9	3.7
**23. I expect to have this illness for the rest of my life**	0.94	0.88	0.89	0.82	0.86	3.3
**24. My illness is a serious condition**	0.8	0.85	0.92	0.89	0.89	3.4
**25. My illness has major consequences on my life**	0.92	0.88	0.88	0.89	0.88	3.3
**26. My illness does not have much effect on my life**	0.82	0.91	0.86	0.83	0.87	3.1
**27. My illness strongly affects the way others see me**	0.89	0.85	0.91	0.83	0.86	3.6
**28. My illness has serious financial consequences**	0.91	0.89	0.89	0.82	0.87	3.6
**29. My illness causes difficulties for those who are close to me**	0.94	0.83	0.9	0.86	0.86	3.3
**30. There is a lot which I can do to control my symptoms**	0.96	0.88	0.85	0.91	0.88	3.8
**31. What I do can determine whether my illness gets better or worse**	0.82	0.91	0.94	0.93	0.93	3.4
**32. The course of my illness depends on me**	0.85	0.88	0.86	0.86	0.87	2.8
**33. Nothing I do will affect my illness**	0.97	0.87	0.89	0.88	0.88	3.1
**34. I have the power to influence my illness**	0.91	0.87	0.91	0.85	0.88	2.8
**35. My actions will have no affect on the outcome of my illness**	0.81	0.91	0.84	0.85	0.87	3.1
**36. My illness will improve in time**	0.96	0.93	0.83	0.92	0.89	3.4
**37. There is very little that can be done to improve my illness**	0.84	0.95	0.84	0.85	0.88	3.5
**38. My treatment will be effective in curing my illness**	0.88	0.95	0.88	0.84	0.89	3.7
**39. The negative effects of my illness can be prevented (avoided) by my treatment**	0.85	0.89	0.88	0.94	0.9	3.1
**40. My treatment can control my illness**	0.91	0.95	0.91	0.92	0.93	3.0
**41. There is nothing that can help my condition**	0.9	0.89	0.86	0.89	0.88	3.5
**42. The symptoms of my condition are puzzling to me**	0.9	0.85	0.81	0.94	0.87	3.9
**43. My illness is a mystery to me**	0.97	0.89	0.85	0.93	0.89	3.9
**44. I don't understand my illness**	0.84	0.9	0.92	0.94	0.92	3.7
**45. My illness doesn't make any sense to me**	0.82	0.9	0.87	0.88	0.88	3.5
**46. I have a clear picture or understanding of my condition**	0.96	0.88	0.9	0.85	0.88	3.1
**47. The symptoms of my illness change a great deal from day to day**	0.84	0.94	0.94	0.91	0.93	3.6
**48. My symptoms come and go in cycles**	0.86	0.9	0.83	0.87	0.87	3.6
**49. My illness is very unpredictable**	0.85	0.9	0.86	0.89	0.88	3.9
**50. I go through cycles in which my illness gets better and worse**	0.96	0.85	0.89	0.83	0.86	3.3
**51. I get depressed when I think about my illness**	0.8	0.92	0.83	0.84	0.86	2.7
**52. When I think about my illness, I get upset**	0.89	0.95	0.88	0.93	0.92	3.2
**53. My illness makes me feel angry**	0.95	0.84	0.88	0.86	0.86	3.0
**54. My illness does not worry me**	0.88	0.92	0.92	0.84	0.89	3.8
**55. Having this illness makes me feel anxious**	0.97	0.88	0.81	0.85	0.85	2.7
**56. My illness makes me feel afraid**	0.89	0.93	0.82	0.84	0.86	3.5
**57. Stress or worry**	0.82	0.93	0.87	0.87	0.89	3.8
**58. Hereditary—it runs in my family**	0.9	0.88	0.91	0.82	0.87	3.1
**59. A germ or virus**	0.87	0.82	0.82	0.85	0.83	2.8
**60. Diet or eating habits**	0.86	0.85	0.89	0.92	0.89	3.1
**61. Chance or bad luck**	0.9	0.93	0.94	0.89	0.92	2.8
**62. Poor medical care in my past**	0.9	0.92	0.86	0.86	0.88	3.8
**63. Pollution in the environment**	0.95	0.83	0.87	0.87	0.86	3.7
**64. My own behavior**	0.87	0.85	0.86	0.88	0.86	3.6
**65. My mental attitude, e.g., thinking about life negatively**	0.81	0.94	0.84	0.91	0.9	3.1
**66. Family problems or worries caused my illness**	0.86	0.91	0.9	0.84	0.88	3.1
**67. Overwork**	0.88	0.84	0.92	0.83	0.86	3.2
**68. My emotional state, e.g., feeling down, lonely, anxious, empty**	0.89	0.92	0.85	0.9	0.89	2.7
**69. Aging**	0.91	0.93	0.86	0.87	0.89	3.4
**70. Alcohol**	0.92	0.82	0.92	0.9	0.88	3.0
**71. Smoking**	0.93	0.85	0.92	0.84	0.87	3.1
**72. Accident or injury**	0.85	0.9	0.9	0.91	0.9	3.3
**73. My personality**	0.93	0.92	0.85	0.94	0.9	3.1
**74. Altered immunity**	0.8	0.95	0.87	0.94	0.92	2.7

#### 3.2.2 Content Validity

All items of the IPQ‐R, as shown in Table 2, had both CVI and CVR greater than 0.80. Furthermore, the qualitative content validity results from the expert panel confirmed the appropriateness of the content, the use of suitable language, adherence to grammatical conventions, and alignment with the scale and its accompanying instructions. As a result, all items were retained for the subsequent steps (Davis 1992;Polit and Beck 2020).

#### 3.2.3 Construct Validity

##### 3.2.3.1 Factor Structure

**FIGURE 1:**
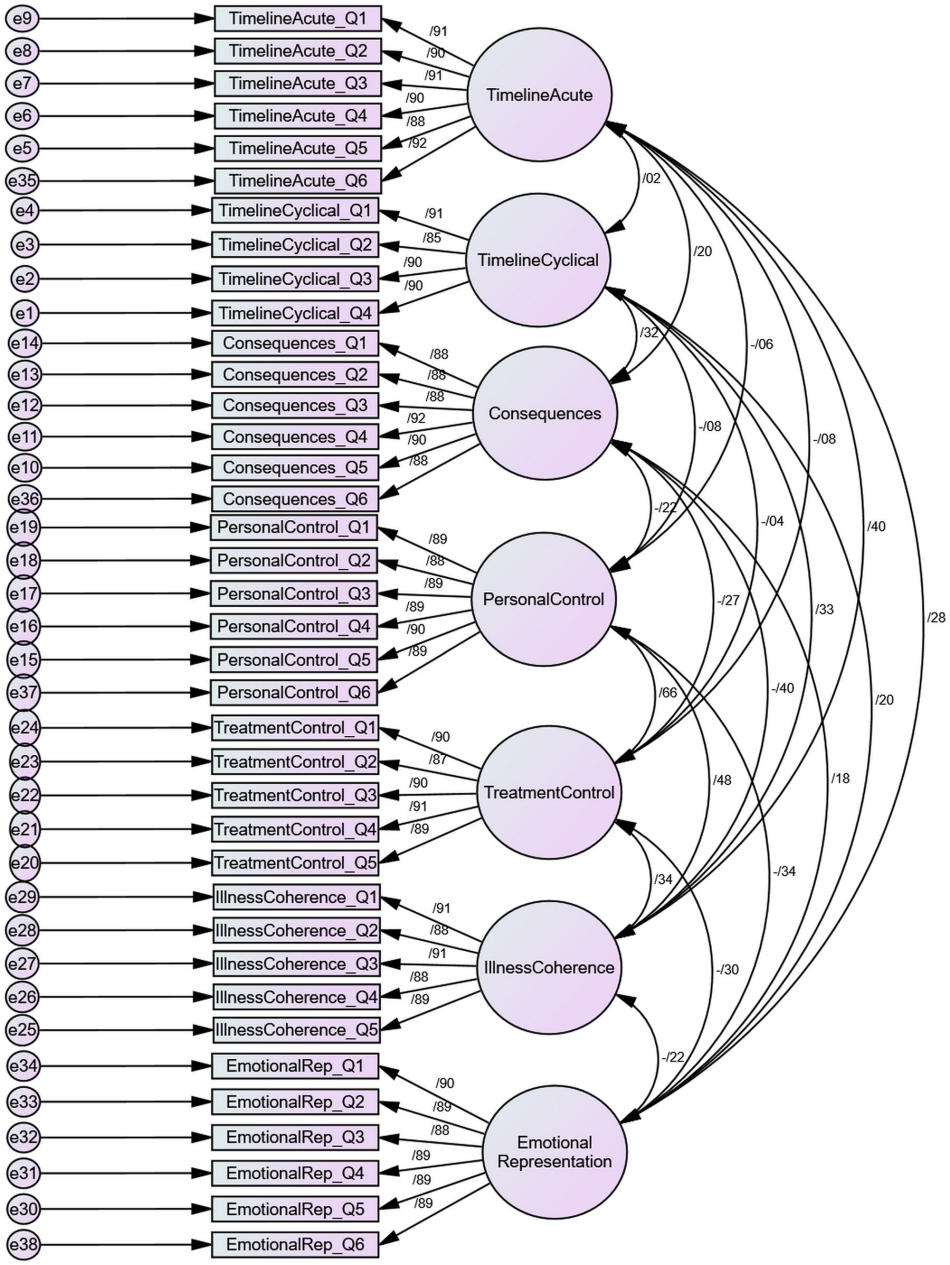
Graphical representation of the confirmatory factor analysis (CFA) model for the Persian version of the IPQ‐R.

The skewness and kurtosis values of the variables included in the CFA ranged from −0.106 to +0.089 and from −0.282 to +0.473, respectively. The maximum Mahalanobis distance was 21.042. Considering that seven observed variables were included in the CFA model, the critical chi‐square value with 7 degrees of freedom at the 0.001 significance level was 24.322. CFA supported the proposed seven‐factor model of the Persian IPQ‐R. The model exhibited excellent fit statistics: *χ* ^2^(644) = 675.008, *χ* ^2^/df = 1.048, CFI = 0.998, SRMR = 0.026, RMSEA = 0.011, and PClose = 1.000. These values fall well within established criteria, indicating a robust factorial structure. A graphical representation of the CFA model and standardized loadings is provided in Figure 1.

##### 3.2.3.2 Structural Validity of Identity Subscale

Paired sample *t*‐test comparing the number of symptoms experienced versus those attributed to MS revealed a significant difference (*t*(399) = −34.91, *p* < 0.001), with participants endorsing more general symptoms (*M* = 0.51) than MS‐attributed ones (*M* = 0.25). The moderate correlation (*r* = 0.458) and large effect size (Cohen's *d* = −1.745) suggest that participants conceptually differentiate between bodily symptoms and illness‐specific attributions. Frequency analysis further supported internal consistency within the subscale, with endorsement rates clustering around 50%, as detailed in Table 3.

**TABLE 3:** Frequency of symptom endorsement and attribution.

Symptom (Q)	Experienced (%)	Attributed (%)
**1. Pain**	50.5	21.8
**2. Sore throat**	51.7	26.3
**3. Nausea**	50.7	24.3
**4. Breathlessness**	50.5	26.8
**5. Weight loss**	54.3	27.0
**6. Fatigue**	49.0	24.0
**7. Stiff joints**	47.0	22.3
**8. Sore eyes**	57.0	28.0
**9. Wheeziness**	49.0	22.8
**10. Headaches**	48.0	21.8
**11. Upset stomach**	52.8	30.3
**12. Sleep difficulties**	50.0	22.0
**13. Dizziness**	54.8	29.3
**14. Loss of strength**	52.0	26.8
**15. Speech distortion**	49.0	22.8
**16. Bladder problems**	49.0	23.3
**17. Clumsiness**	49.5	24.5
**18. Numbness**	49.3	27.3

##### 3.2.3.3 Structure of Cause Subscale

The skewness and kurtosis values of the variables included in the EFA ranged from −0.007 to +0.482 and from −1.112 to −0.263, respectively. The maximum Mahalanobis distance was 12.699. Considering that 18 observed variables were included in the EFA model, the critical chi‐square value with 14 degrees of freedom at the 0.001 significance level was 43.82. The KMO statistic was 0.904, and Bartlett's test of sphericity was significant (*χ* ^2^ = 5940.019, df = 153, *p* < 0.001). Four factors were extracted on the basis of eigenvalues greater than 1.0 and scree plot inspection, collectively accounting for 75.6% of the total variance after rotation. The extracted factors were thematically labeled as follows: Factor 1, internal and immunological causes (e.g., “my personality” and “changes in my immune system”); Factor 2, psychosocial and lifestyle factors (e.g., “negative thinking,” “emotional state,” “family problems,” “aging,” and “pollution”); Factor 3, biological and situational risk factors (e.g., “stress,” “heredity,” “viral infections,” “diet,” “bad luck,” and “past medical care”); and Factor 4, risk behaviors and physical trauma (e.g., “alcohol,” “smoking,” and “accident or injury”). No substantial cross‐loadings were observed, and all factor loadings exceeded the recommended cutoff of 0.40. Communalities ranged from 0.517 to 0.999, with the majority of items demonstrating strong shared variance with the extracted factors. However, one communality estimate exceeded 1.0 (immunity_Q3), possibly due to iteration artifacts. Detailed factor structure and communalities are presented in Table 4.

**TABLE 4:** Rotated component matrix for the causal attribution subscale.

Item	Component 1	Component 2	Component 3	Component 4
**57. Stress or worry**		0.851		
**58. Hereditary—it runs in my family**		0.864		
**59. A germ or virus**		0.865		
**60. Diet or eating habits**		0.838		
**61. Chance or bad luck**		0.857		
**62. Poor medical care in my past**		0.875		
**63. Pollution in the environment**	0.879			
**64. My own behavior**	0.888			
**65. My mental attitude, e.g., thinking about life negatively**	0.866			
**66. Family problems or worries caused my illness**	0.867			
**67. Overwork**	0.875			
**68. My emotional state, e.g., feeling down, lonely, anxious, empty**	0.885			
**69. Aging**	0.869			
**70. Alcohol**			0.868	
**71. Smoking**			0.838	
**72. Accident or injury**			0.872	
**73. My personality**				0.718
**74. Altered immunity**				0.998

Most theoretically related subscales were moderately to strongly correlated. For instance, timeline cyclical and consequences demonstrated a correlation of *r* = 0.302. A detailed summary of these results is provided in Table 5.

**TABLE 5:** Correlation matrix of Revised Illness Perception Questionnaire (IPQ‐R) subscales.

Subscale	Internal and immunological	Psychosocial and lifestyle factors	Biological and situational risk	Risk behaviors and physical trauma	Timeline acute	Timeline cyclical	Consequences	Personal control	Treatment control	Illness coherence	Emotional representation	Identity
**Internal and immunological**	1											
**Psychosocial and lifestyle factors**	−0.139^**^	1										
**Biological and situational risk**	0.008	−0.001	1									
**Risk behaviors and physical trauma**	0.011	0.056	−0.004	1								
**Timeline acute**	0.051	0.000	0.006	0.058	1							
**Timeline cyclical**	−0.028	0.073	−0.009	−0.021	0.016	1						
**Consequences**	0.069	0.023	0.018	0.003	0.198^**^	0.302^**^	1					
**Personal control**	−0.014	−0.031	−0.007	−0.013	−0.062	−0.072	−0.212^**^	1				
**Treatment control**	−0.054	0.041	−0.027	0.031	−0.073	−0.044	−0.259^**^	0.631^**^	1			
**Illness coherence**	0.031	−0.030	−0.014	−0.031	0.378^**^	0.312^**^	−0.388^**^	0.455^**^	0.324^**^	1		
**Emotional representation**	0.006	0.019	−0.045	0.074	0.270^**^	0.191^**^	0.177^**^	−0.324^**^	−0.288^**^	−0.209^**^	1	
**Identity**	−0.024	0.034	−0.043	0.041	0.272^**^	0.350^**^	0.505^**^	−0.344^**^	−0.303^**^	−0.181^**^	0.431^**^	1

* indicates significance at *p* < 0.05, and ** indicates significance at *p* < 0.01.

The AVE for the Identity subscale was notably low at 0.077, indicating insufficient convergent validity. In contrast, the AVE values for the other subscales ranged from 0.739 to 1.071. Full details of this analysis are presented in Table 6.

**TABLE 6:** Average variance extracted (AVE), maximum shared variance (MSV), and square root of AVE (√AVE) for each Revised Illness Perception Questionnaire (IPQ‐R) subscale.

Subscale	AVE	MSV	√AVE
**Timeline cyclical**	0.795	0.227	0.892
**Timeline acute**	0.813	0.158	0.902
**Consequences**	0.791	0.461	0.889
**Personal control**	0.792	0.440	0.890
**Treatment control**	0.798	0.440	0.893
**Illness coherence**	0.798	0.226	0.893
**Emotional representation**	0.793	0.316	0.891
**Identity**	0.077	0.461	0.277
**Internal and immunological**	0.740	0.021	0.860
**Psychosocial and lifestyle factors**	0.770	0.021	0.877
**Biological and situational risk**	0.739	0.002	0.860
**Risk behaviors and physical trauma**	1.071	1.121	1.034

Discriminant validity was generally supported. Correlations between conceptually distinct subscales were low; for example, psychosocial and lifestyle factors and biological and situational risk had a near‐zero correlation (*r* = −0.001). Further details are available in Table 5.

Moreover, most HTMT values were below the conservative threshold of 0.85; for instance, the HTMT value between risk behaviors and physical trauma and timeline cyclical was 0.317. Detailed results of this analysis are presented in Table 7.

**TABLE 7:** Heterotrait–Monotrait (HTMT) ratio of correlations between Revised Illness Perception Questionnaire (IPQ‐R) subscales.

Subscale	Internal and immunological	Psychosocial and lifestyle factors	Biological and situational risk	Risk behaviors and physical trauma	Timeline acute	Timeline cyclical	Consequences	Personal control	Treatment control	Illness coherence	Emotional representation	Identity
**Internal and immunological**	—											
**Psychosocial and lifestyle factors**	0.146	—										
**Biological and situational risk**	0.009	0.003	—									
**Risk behaviors and physical trauma**	0.012	0.062	0.005	—								
**Timeline acute**	0.054	0.001	0.017	0.066	—							
**Timeline cyclical**	0.028	0.078	0.005	0.023	0.015	—						
**Consequences**	0.074	0.025	0.028	0.004	0.207	0.317	—					
**Personal control**	0.014	0.032	0.006	0.014	0.064	0.076	0.221	—				
**Treatment control**	0.057	0.043	0.024	0.036	0.076	0.047	0.271	0.661	—			
**Illness coherence**	0.035	0.030	0.009	0.033	0.392	0.330	0.407	0.477	0.340	—		
**Emotional representation**	0.007	0.020	0.042	0.084	0.281	0.202	0.185	0.337	0.301	0.220	—	
**Identity**	0.032	0.039	0.032	0.073	0.353	0.470	0.678	0.465	0.402	0.255	0.565	—

### 3.3 Reliability

For the subscales assessed using CFA, both Cronbach's alpha coefficients (ranging from 0.939 to 0.962) and CR values (ranging from 0.939 to 0.963) were calculated, indicating excellent internal consistency. For the subscales analyzed through EFA and the Identity subscale, only Cronbach's alpha was reported. The four‐cause subscales showed acceptable to excellent reliability (*α* = 0.839–0.959), whereas the identity subscale demonstrated lower internal consistency (*α* = 0.590). Detailed results are presented in Table 8.

**TABLE 8:** Cronbach's alpha and composite reliability (CR) of Revised Illness Perception Questionnaire (IPQ‐R) subscales.

Subscale	Cronbach's alpha	Composite reliability (CR)
**Timeline acute/Chronic**	0.962	0.963
**Timeline cyclical**	0.939	0.939
**Consequences**	0.958	0.958
**Personal control**	0.958	0.958
**Treatment control**	0.951	0.952
**Illness coherence**	0.951	0.952
**Emotional representation**	0.958	0.958
**Identity**	0.590	—
**Internal and immunological**	0.945	—
**Psychosocial and lifestyle factors**	0.959	—
**Biological and situational risk**	0.895	—
**Risk behaviors and physical trauma**	0.839	—

## 4 Discussion

This study evaluated the psychometric properties of the Persian version of the IPQ‐R in a sample of Iranian MS patients. The validation process involved thorough assessments of face and content validity, construct validity (via EFA and CFA), and internal consistency reliability. The findings confirmed the multidimensional structure of the IPQ‐R and its suitability for Iranian MS patients. Most subscales exhibited robust psychometric properties, though the identity subscale revealed limitations requiring further refinement. This study enhances understanding of illness perception in MS and provides a reliable tool for clinical and research purposes.

### 4.1 Face and Content Validity

The Persian IPQ‐R demonstrated strong face and content validity. Expert panels and patients found all items relevant, clear, and comprehensible. Content validity indices (CVI and CVR) surpassed acceptable thresholds, affirming the items’ ability to capture intended constructs. These results align with validation studies in other contexts, such as the Chinese IPQ‐R for hypertension (Chen et al. 2008), stress urinary incontinence (SUI) (Fan et al. 2017), the Swedish version for post‐myocardial infarction patients (Brink et al. 2011), and the Polish version for cancer patients (Pasternak et al. 2021).

### 4.2 Construct Validity

CFA results supported the original seven‐factor model of the IPQ‐R proposed by Moss‐Morris et al. (2002), including the subscales of timeline (acute/chronic), timeline cyclical, consequences, personal control, treatment control, illness coherence, and emotional representation. The model showed acceptable fit in the Iranian MS sample, confirming the structural integrity of the Persian IPQ‐R.

This finding aligns with the majority of international validation studies of the IPQ‐R across diverse medical and cultural contexts. For instance, the Chinese version for nasopharyngeal carcinoma (NPC) (Cai et al. 2023), the Swedish version for post‐myocardial infarction patients (Brink et al. 2011), the Chinese versions for obstructive sleep apnea (OSA) (Yu et al. 2023) and breast cancer–related lymphedema (BCRL) (Huang et al. 2019), the Spanish version for dental caries (Villalobos‐Galvis et al. 2017), the Polish version for cancer (Pasternak et al. 2021), and studies on end‐stage renal disease (ESRD) (Chilcot et al. 2012) and sickle cell disease (Oudin Doglioni et al. 2022) all reported acceptable or strong support for the seven‐factor structure, often with minimal item modifications.

However, some discrepancies were observed in other studies, including those conducted among patients with SUI (Fan et al. 2017), internet gaming disorder (Lau et al. 2020), Type 2 diabetes and cardiovascular disease (Brzoska et al. 2012), and parents of children with autism spectrum disorder (ASD) (Mire et al. 2018). In these cases, the seven‐factor structure was either not fully supported or required substantial modifications, such as item deletion, error covariance adjustments, or structural reconfiguration. Common contributing factors to these inconsistencies include differences in cultural interpretation, disease‐specific symptomatology, and potential issues with reverse‐worded items.

The findings of the present study provide empirical support for the construct validity of the Identity subscale within the IPQ‐R, particularly through the demonstration of known‐group validity. Participants significantly differentiated between general bodily symptoms and those they attributed specifically to MS, as reflected by a large effect size and a moderate correlation.

These results suggest that individuals form distinct cognitive representations regarding symptom attribution. This reinforces the conceptual basis of the illness identity construct. The findings are in line with previous validation studies that highlight the uniqueness of the identity subscale.

For instance, a Polish study involving cancer patients used Wilcoxon signed‐rank tests and found significant differences between experienced symptoms and those attributed to the illness (Pasternak et al. 2021). Similarly, a study of German patients with schizophrenia showed moderate correlations between the identity subscale and clinical insight measures. Although these correlations often fall below the conventional threshold for strong convergent validity (*r* ≥ 0.70), they still indicate that the subscale captures a distinct aspect of illness perception (Cavelti et al. 2012).

Additionally, frequency analysis in the current study revealed a coherent pattern of symptom endorsement. Certain MS‐related symptoms were consistently attributed to the illness by participants. Although this pattern does not imply the existence of a unidimensional latent factor, it reflects internal coherence in responses. Such within‐group consistency further supports the meaningfulness of the identity subscale's content and mirrors findings from other IPQ‐R validation efforts.

The results of the current study support the construct validity of the cause subscale of the IPQ‐R in individuals with MS. Using EFA, four interpretable and theoretically meaningful factors were extracted: internal and immunological causes (Khutsishvili et al. 2024), psychosocial and lifestyle factors (Mirmosayyeb et al. 2022), biological and situational risk factors (Ziaie et al. 2024), and risk behaviors and physical trauma (Wilski et al. 2019). Together, these factors accounted for over 75% of the variance, indicating a well‐defined underlying structure.

This structure partially aligns with the four causal dimensions proposed by Moss‐Morris et al. (2002): psychological attributions, risk factors, immunity, and chance (Moss‐Morris et al. 2002). However, in the current study, internal causes (e.g., personality traits) and immunological causes were grouped into a single factor. This pattern may reflect illness‐specific representations among MS patients, for whom immune dysregulation plays a central explanatory role. Such divergence from the original model highlights the contextual nature of causal beliefs and supports the original suggestion by the scale developers that the factor structure of this subscale may vary across illness types.

The factor structure identified in this study is also consistent with findings from other illness‐specific validation studies. For example, in samples with ESRD (Chilcot et al. 2012), hypertension (Chen et al. 2008), and chronic conditions in North African countries such as Algeria (Aberkane 2017), similar multi‐factorial structures were reported. In these contexts, causal beliefs were often categorized into biologically based, psychosocial, behavioral, and external domains. Nevertheless, the precise composition of these factors tends to differ depending on the cultural and medical context.

Cultural specificity is particularly evident in Chinese studies on cervical cancer (Chen et al. 2020) and BCRL (Huang et al. 2019), for example, where five distinct causal dimensions emerged. These included beliefs related to sexuality, fertility, and environmental causes—dimensions shaped by sociocultural narratives and local understandings of illness.

In contrast, some studies—especially those in dermatology (e.g., atopic dermatitis) (Wittkowski et al. 2008) or oncology (e.g., Polish cancer patients) (Pasternak et al. 2021)—failed to establish a stable factor structure. In these cases, low internal consistency prompted researchers to recommend that causal items be treated as standalone indicators, rather than as part of a unified scale.

This variability is expected, as causal beliefs are shaped by a complex interplay of biomedical knowledge, cultural values, personal experiences, and sources of health information. Therefore, the structure of the Cause subscale should be viewed as fluid and context‐dependent, rather than universally fixed.

The findings of the present study provide compelling evidence for the convergent and discriminant validity of the Persian version of the IPQ‐R in individuals with MS. Moderate‐to‐strong correlations were observed among conceptually related subscales. For example, a significant association between “timeline cyclical” and “consequences” supports the notion that the instrument adequately captures theoretically meaningful constructs.

These findings are consistent with prior validation studies of the IPQ‐R in other clinical populations. In patients with cancer‐related fatigue (CRF) (Pertl et al. 2012), OSA (Yu et al. 2023), and schizophrenia (Cavelti et al. 2012), similar inter‐subscale correlation patterns were found. Notably, strong associations between the “identity” and “consequences” subscales and indicators of symptom burden or emotional distress were reported—such as correlation coefficients ranging from 0.70 to 0.78 in CRF studies (Pertl et al. 2012).

Most subscales achieved AVE values above 0.50, aligning with validations for BCRL (Huang et al. 2019), NPC (Cai et al. 2023), and SUI (Fan et al. 2017). Yet, the markedly low AVE for the “identity” subscale (AVE = 0.077) in our sample may indicate variation in symptom interpretation among MS patients, which warrants further qualitative investigation.

In terms of discriminant validity, low correlations between theoretically unrelated constructs and HTMT values below the conservative 0.85 threshold offer robust support for the distinctiveness of the IPQ‐R dimensions.

For example, the near‐zero correlation between “psychosocial and lifestyle factors” and “biological and situational risk” (*r* = −0.001) and low HTMT values, such as between “risk behaviors and physical trauma” and “timeline cyclical” (HTMT = 0.317), confirm that the subscales are measuring distinct psychological representations. This finding is in line with the SUI (Fan et al. 2017) and Type 2 diabetes (Shiyanbola et al. 2022) studies, which reported low inter‐factor correlations (*r* < 0.50) and used similar statistical methods, including the Fornell–Larcker criterion and HTMT ratio, to confirm discriminant validity.

The four‐factor structure of the cause subscale also showed strong discriminant properties, with each factor exhibiting minimal overlap with the others. This multidimensional pattern corresponds with causal belief structures reported in other chronic illnesses, such as ESRD (Chilcot et al. 2012), hypertension (Al‐Ghamdi et al. 2022), and cervical cancer (Chen et al. 2020), highlighting the influence of sociocultural and illness‐specific contexts on attribution models.

In contrast, several prior studies encountered challenges in establishing discriminant validity. For example, in research involving individuals with ASD (Mire et al. 2018) and mild traumatic brain injury (MTBI) (Snell et al. 2010), the personal control and treatment control subscales failed to emerge as distinct constructs, indicating poor discriminant validity in those contexts.

Similarly, in the case of substance dependence, the original factor structure showed poor model fit, likely due to participants’ non‐disease attribution of addiction, compromising the theoretical clarity required for construct differentiation (Mo et al. 2015).

Further, other studies, such as those on ESRD, had to revise specific items to enhance the separation between control‐related factors. In particular, the removal of two items and reassignment of another (Item 23) improved the model's discriminant capacity (Chilcot et al. 2012). A Turkish version applied to patients with diabetes and cardiovascular disease also failed to confirm the original IPQ‐R structure without major modifications, suggesting significant cultural and clinical variability in how illness perceptions manifest across populations (Brzoska et al. 2012).

### 4.3 Reliability

The present study evaluated the reliability of the Persian version of the IPQ‐R in an Iranian sample of individuals with MS. The findings provide compelling evidence for the high internal consistency and CR of most subscales, particularly those confirmed via CFA, with Cronbach's alpha and CR values ranging from 0.939 to 0.962. These results align with the psychometric properties reported by Moss‐Morris et al. (2002), the developers of the original IPQ‐R, who documented good internal consistency (*α* = 0.79–0.89) and test–retest reliability across various illness dimensions (Moss‐Morris et al. 2002).

However, the identity subscale in the present study demonstrated weak reliability (Cronbach's alpha = 0.590), which mirrors findings from multiple cross‐cultural validations. For instance, Chen et al. (2008) reported a similarly low alpha (0.57) for the identity subscale in Chinese patients with hypertension (Chen et al. 2008), and Huang et al. (2019) found an alpha of 0.61 in a sample of Chinese women with BCRL (Huang et al. 2019). Additionally, some studies chose not to evaluate the internal consistency of the identity subscale at all (Cavelti et al. 2012; Wittkowski et al. 2008; Zhou et al. 2020), citing its conceptual role as a symptom checklist rather than a coherent latent construct. These findings suggest that the identity subscale may not function consistently across different cultural or clinical populations.

In the current study, the weak internal consistency may reflect the heterogeneous symptom experiences of Iranian patients with MS or variability in the attribution of listed symptoms to the illness. This supports the argument that the symptom items in the identity subscale should be reviewed and possibly revised for cultural and illness‐specific relevance.

Nevertheless, it is worth noting that a few studies have reported acceptable reliability for the identity subscale. For instance, Fan et al. (2017) and Pasternak et al. (2021) both found *α* = 0.83 in different clinical populations, suggesting that the psychometric performance of this subscale may be context‐dependent (Fan et al. 2017; Pasternak et al. 2021).

In contrast, other subscales in the present study showed excellent reliability, outperforming or matching results reported in previous studies. For example, although the treatment control subscale showed only moderate reliability in prior research (e.g., *α* = 0.67 in Chen et al. 2008; CR = 0.638 in Chen et al. 2020), it demonstrated high reliability in the current sample (Chen et al. 2008; Chen et al. 2020). Similarly, the personal control subscale, which showed modest alphas in studies by (Chen et al. 2020) and (Fan et al. 2017), performed substantially better here (Chen et al. 2020) and (Fan et al. 2017).

Subscales such as timeline cyclical, consequences, illness coherence, and the four‐cause dimensions also displayed strong internal consistency, exceeding the results of some international studies, where these subscales were occasionally reported as weaker (Albert et al. 2013; Mire et al. 2018; Pasternak et al. 2021; Surgenor et al. 2020; Snell et al. 2010; Wittkowski et al. 2008).

### 4.4 Practical Implications

The availability of a valid and reliable Persian version of the IPQ‐R for Iranian MS patients offers significant clinical advantages. It enables healthcare providers to identify maladaptive illness beliefs that may hinder treatment adherence or negatively affect quality of life.

Using the IPQ‐R, tailored psychoeducational interventions can be developed to target specific misconceptions—such as beliefs in uncontrollability or treatment ineffectiveness. Addressing these beliefs may enhance patients’ illness coherence and improve their coping strategies.

Moreover, the instrument can promote more empathetic and patient‐centered communication. By aligning treatment plans with patients’ beliefs and values, clinicians can foster a more collaborative therapeutic relationship.

The IPQ‐R also offers utility in evaluating the effectiveness of clinical or rehabilitation interventions from the patient's perspective. Additionally, by assessing perceived levels of personal and treatment control, the tool can inform the design of self‐management programs that empower patients to take a more active role in managing their condition.

### 4.5 Research Implications

This study lays the groundwork for future research in several directions. Longitudinal studies can explore how illness perceptions evolve across different stages of MS and their relationship with outcomes such as fatigue, disability progression, or treatment adherence. The validated instrument enables development and evaluation of targeted interventions aiming to reshape maladaptive illness beliefs.

### 4.6 Strengths and Limitations

This study presents several key strengths. Most notably, it offers a comprehensive psychometric evaluation of the Persian version of the IPQ‐R among Iranian patients with MS. The assessment covers face, content, and construct validity—examined through both EFA and CFA—as well as convergent and discriminant validity, using AVE, HTMT ratios, and inter‐subscale correlations.

In addition, reliability was thoroughly evaluated using Cronbach's alpha and CR indices. This multifaceted approach enhances confidence in the tool's clinical and research applications, establishing a solid foundation for its use in Persian‐speaking MS populations.

Despite these strengths, several limitations should be acknowledged. First, the cross‐sectional design—conducted between December 2023 and April 2024—limits the ability to assess changes in illness perception over time or draw causal inferences between illness beliefs and clinical outcomes. Future longitudinal research is needed to examine how perceptions evolve across different stages of MS.

Second, the use of convenience sampling may restrict the generalizability of the findings. Participants were recruited from a specific MS community in Iran, which may not fully represent the broader MS population with diverse sociodemographic and clinical profiles.

Finally, although the Persian IPQ‐R demonstrated strong psychometric properties, full normative validation has not yet been completed. Further studies involving larger and more heterogeneous samples from various regions and healthcare contexts are necessary to establish normative data and enhance the tool's broader applicability.

Addressing these limitations in future research will strengthen the reliability and generalizability of the Persian IPQ‐R and support its use across clinical and research settings within the Iranian MS community.

## 5 Conclusion

The Persian version of the IPQ‐R demonstrated acceptable psychometric properties among Iranian patients with MS. The seven‐factor structure was supported, and most subscales showed satisfactory reliability and validity. Although the identity subscale exhibited limitations, the overall findings affirm the utility of the instrument for both research and clinical assessment of illness perceptions in this population.

## Author Contributions

**Ali Bagheri**: conceptualization, writing – original draft, formal analysis, methodology, validation, resources. **Maryam Amini‐Fasakhoudi**: data curation, project administration, conceptualization. **Mohammad Ali Sahraian**: supervision. **Abbas Masjedi‐Arani**: supervision, writing – review and editing, investigation. **Mohadeseh Hashemi**: data curation. **Fatemeh Serjouie**: data curation. **Amir Sam Kianimoghadam**: supervision, writing – review and editing, investigation.

## Conflicts of Interest

The authors declare no conflicts of interest.

## Peer Review

The peer review history for this article is available at https://publons.com/publon/10.1002/brb3.70821.

## Additional Information

Correspondence and requests for materials should be addressed to Amir Sam Kianimoghadam.

## Data Availability

The data supporting this study's fndings are available from the corresponding author upon reasonable request.
